# Polyandry Is a Common Event in Wild Populations of the Tsetse Fly *Glossina fuscipes fuscipes* and May Impact Population Reduction Measures

**DOI:** 10.1371/journal.pntd.0001190

**Published:** 2011-06-07

**Authors:** Angelica Bonomi, Federico Bassetti, Paolo Gabrieli, Jon Beadell, Marco Falchetto, Francesca Scolari, Ludvik M. Gomulski, Eugenio Regazzini, Johnson O. Ouma, Adalgisa Caccone, Loyce M. Okedi, Geoffrey M. Attardo, Carmela R. Guglielmino, Serap Aksoy, Anna R. Malacrida

**Affiliations:** 1 Department of Animal Biology, University of Pavia, Pavia, Italy; 2 Department of Mathematics, University of Pavia, Pavia, Italy; 3 Department of Ecology and Evolutionary Biology, Yale University, New Haven, Connecticut, United States of America; 4 Trypanosomiasis Research Centre, Kenya Agricultural Research Institute, Kikuyu, Kenya; 5 National Livestock Resources Research Institute, Tororo, Uganda; 6 Division of Epidemiology of Microbial Diseases, Yale School of Public Health, New Haven, Connecticut, United States of America; 7 Department of Genetics and Microbiology, University of Pavia, Pavia, Italy; IRD/CIRDES, Burkina Faso

## Abstract

**Background:**

*Glossina fuscipes fuscipes* is the main vector of human and animal trypanosomiasis in Africa, particularly in Uganda. Attempts to control/eradicate this species using biological methods require knowledge of its reproductive biology. An important aspect is the number of times a female mates in the wild as this influences the effective population size and may constitute a critical factor in determining the success of control methods. To date, polyandry in *G.f. fuscipes* has not been investigated in the laboratory or in the wild. Interest in assessing the presence of remating in Ugandan populations is driven by the fact that eradication of this species is at the planning stage in this country.

**Methodology/Principal Findings:**

Two well established populations, Kabukanga in the West and Buvuma Island in Lake Victoria, were sampled to assess the presence and frequency of female remating. Six informative microsatellite loci were used to estimate the number of matings per female by genotyping sperm preserved in the female spermathecae. The direct count of the minimum number of males that transferred sperm to the spermathecae was compared to *Maximum Likelihood* and *Bayesian* probability estimates. The three estimates provided evidence that remating is common in the populations but the frequency is substantially different: 57% in Kabukanga and 33% in Buvuma.

**Conclusions/Significance:**

The presence of remating, with females maintaining sperm from different mates, may constitute a critical factor in cases of re-infestation of cleared areas and/or of residual populations. Remating may enhance the reproductive potential of re-invading propagules in terms of their effective population size. We suggest that population age structure may influence remating frequency. Considering the seasonal demographic changes that this fly undergoes during the dry and wet seasons, control programmes based on SIT should release large numbers of sterile males, even in residual surviving target populations, in the dry season.

## Author Summary

*Glossina fuscipes fuscipes* is the most common tsetse species in Uganda where it is responsible for transmitting *Trypanosoma brucei rhodensiense* and *Trypanosoma brucei gambiense* parasites causing sleeping sickness in humans in addition to related trypanosomes that cause Nagana in cattle. An understanding of the reproductive biology of this vector is essential for the application of sustainable control/eradication methods such as Sterile Insect Technique (SIT). We have analysed the number of times a female mates in the wild as this aspect of the reproductive behaviour may affect the stability and size of populations. We provide evidence that remating is a common event in the wild and females store sperm from multiple males, which may potentially be used for insemination. In vector eradication programmes, re-infestation of cleared areas and/or in cases of residual populations, the occurrence of remating may unfortunately enhance the reproductive potential of the re-invading propagules. We suggest that population age structure may influence remating frequency. Considering the seasonal demographic changes that this fly undergoes during the dry and wet seasons, control programmes based on SIT should release large numbers of sterile males, even in residual surviving target populations, in the dry season.

## Introduction

Tsetse flies (Diptera: Glossinidae) are the sole vectors of pathogenic trypanosomes in tropical Africa, where they cause Human African Trypanosomiasis (HAT), or sleeping sickness, one of the most seriously neglected tropical diseases. HAT is a zoonosis caused by the flagellate protozoa *Trypanosoma brucei rhodesiense* in East and Southern Africa and by *T. b. gambiense* in West and Central Africa [1]. The only country with known infection foci of both parasites is Uganda [2]. The World Health Organization (WHO) has estimated that there are around 10,000 cases of HAT as the recent epidemics are beginning to decline, but 60 million people continue to live at risk in 37 countries covering about 40% of Africa [3]. In addition to HAT, trypanosomes transmitted by tsetse cause a fatal disease in livestock, called Nagana, which represents a major impediment to agricultural development in Africa. No vaccines exist to prevent the disease and drugs currently available to treat HAT are expensive, can cause severe side-effects, and are difficult to administer in remote villages. As a consequence, an effective alternative for controlling the disease is to target the tsetse vector [1], [4]. In 2001, the African Union launched the Pan African Tsetse and Eradication Campaign (PATTEC) to increase efforts to manage this plague, which is considered one of the root causes of hunger and poverty in most sub-Saharian African countries [5].

*Glossina fuscipes fuscipes*, a member of the *palpalis* complex, is one of the most important vectors of human and animal trypanosomiasis in Africa. It is a riverine species confined to forested patches along rivers and lacustrine environments [6]. Its range extends across the central part of the African continent from Sudan, Democratic Republic of Congo to Uganda. As a trypanosome vector, *G. f. fuscipes* is exposed to a large reservoir of parasites, as it feeds on both domestic and wild animals in addition to humans.

Attempts to control/eradicate tsetse require in-depth information about their population characteristics such as dispersal rates, distribution, densities and reproductive biology. The riverine nature of *G. f. fuscipes* has resulted in a patchy distribution of its populations and as a consequence of drift, populations arising from historical colonization events show a considerable population structure [7]. Nevertheless, Beadell et al. [8] inferred a high dispersal capacity for *G. f. fuscipes*, demonstrating ongoing gene flow among apparently isolated populations, with an equilibrium between drift and gene flow in western and south-eastern Uganda. Since populations undergo seasonal contractions during the year due to changes in water availability, Krafsur [9] suggests that high levels of genetic drift during the dry season could be masking effects due to gene flow.

The capacity of *G. f. fuscipes* to disperse and colonize may also depend on the number of times a female mates in the wild and whether the matings are with the same or different males. This specific mating behaviour influences the effective population size, and may constitute a critical factor in determining the success of control methods [10], [11]. Some aspects of mating behaviour, such as the effect of age on mating competitiveness, have been studied in laboratory colonies [12], but to date, the polyandrous behaviour of *G. f. fuscipes* has not investigated in the laboratory or in the wild.

Data on the proportion of tsetse females that mate more than once can be obtained in two ways: through the number of fathers (male genotype) represented in her offspring [13], [14] or through genotyping stored sperm in the spermatheca of the female. In the first case, the genotyping of offspring can reveal the minimum number of males that sire a brood, but not necessarily the number of males with which a female had mated, as females may bias paternity towards one or a few of their mates, resulting in an underestimation of the actual level of polyandry [15]. In the second case a more accurate estimate of the number of mates can be obtained, through the genotyping of the female's stored sperm supply [16], [17].

Using microsatellite markers to genotype sperm, we ascertained the minimum number of males that were able to transfer sperm to a female's spermatheca in two Uganda populations. The interest in Uganda is based on the fact that eradication efforts by PATTEC are at the planning stages in this country. The results obtained in two sites, which are eco-geographically differentiated, are of particular interest, as in both populations a large proportion of females were found to have mated more than once. The remating frequencies, validated with probability values obtained with two inference statistical models, are relevant for interpreting the reproductive biology of the species but may also have an immediate impact on the strategy to be employed for eradication success.

## Materials and Methods

### Study sites and sampling

**Figure 1:**
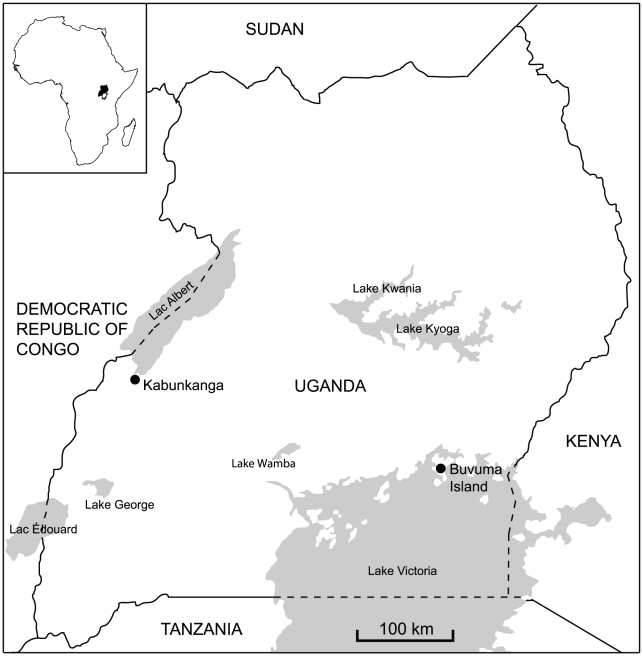
Map of the two sampling locations of *Glossina fuscipes fuscipes* in Uganda.

Natural populations of *G. f. fuscipes* were sampled from two localities in Uganda: Kabunkanga (KB, Western territory, 0°58′37.88″N, 30°32′47.40″E) and Buvuma Island in Lake Victoria (BV, Southern zone, 0°15′23.15″N, 33°12′22.86″E) (Figure 1). Both sites are favourable for this riverine species and harbour well established populations. Males and females were collected using biconical traps located 500 m apart at both sites. The traps were checked daily and the average daily fly catch per trap was recorded. The collections from Kabunkanga were made in November 2008, at the end of the dry season from four traps with an average of 15 flies/day/trap. The collections from Buvuma Island were made at the beginning of April 2008, during the wet season, from five traps with an average of 58 flies/day/trap. Individuals of each sex were removed from the traps and placed in tubes containing 95% ethanol. The Kabunkanga (KB) sample was composed of 20 males and 29 females, while for Buvuma Island (BV) 20 males and 40 females were analyzed. The number of males and females in each sample mirrored the sex-ratio observed in the collections. The age and the reproductive history of the sampled flies were unknown, but all the 29 Kabukanga females and the 40 females collected in Buvuma had mated as their spermathecae contained sperm. More precise information about the age structure of the flies collected in each sample could have been obtained from ovarian inspection and/or wing fray analysis [18]; however the extent of damage observed in the wings due to trapping and EtOH preservation, did not permit wing fray analysis. Ovarian age was not assessed. For each site, all of the collected flies were considered to compute allele frequencies and variability estimates. For the remating analysis, the 29 females from KB and 30 females, randomly chosen from the BV collection, were examined.

### Sperm isolation and DNA extraction

For sperm isolation, the ethanol preserved female body was rehydrated in physiological solution (0.9% NaCl) for 24–48 h before dissection. The spermathecae were easily isolated from the abdomen, stored in 70% ethanol to permit the sperm to coagulate in a “sperm bundle” [19] and then dissected in a drop of 1× PBS (Phosphate buffered saline). The sperm bundle was isolated and DNA extraction was performed using QIAamp DNA Micro Kit (Qiagen, Valencia, CA). DNA extraction from the legs was performed using the protocol described in Baruffi et al. [20]. The DNA extracted from legs and sperm was used as PCR template for the amplification of microsatellite markers (SSRs).

### Microsatellite characterization

Nineteen SSR loci were previously isolated from a *G. f. fuscipes* SSR enriched library [7]. For eight of these loci (A06, A09, A112, B05, C7, C107, D06, and D109) the described primer sequences were adopted [7], [8], [21]. For the remaining 11 loci (A03, B03, B06, B11, B109, C104, D3, D05, D12, D101 and D103) primer sequences and amplification conditions were determined using DNA extracted from Kabunkanga flies as PCR template. Amplification reactions were performed in 15 µl volumes containing 1 µl of genomic DNA, 1× reaction buffer, 1.5 mM MgCl_2_, 25 µM dNTP, 1 U Taq polymerase (Invitrogen, Carlsbad, CA) and 10 pmol of each primer. Reactions were performed with an Eppendorf MasterCycler Gradient thermocycler. After an initial denaturing step of 10 min at 96°C, the PCR consisted of 40 cycles of 1 min at 96°C, 1 min at optimal annealing temperature, and 1 min at 72°C, followed by a final extension step of 15 min at 72°C. Microsatellite loci were analyzed using an ABI PRISM 310 Genetic Analyzer and the GeneScan program (Applied Biosystems). An individual was declared null (non-amplifying allele) after at least two amplification failures.

### Microsatellite chromosomal location by *in situ* hybridization

Mitotic chromosome spreads were obtained from freshly deposited larvae obtained from the Slovakia laboratory strain. Briefly, brain tissues were incubated in 1% sodium citrate for 10 min at room temperature and transferred to methanol-acetic acid 3∶1 solution for 4 min. The material was disrupted in 100 µl 60% acetic acid and dropped onto clean slides and dried. Pre-hybridization was performed according to Willhoeft [22]. *In situ* hybridization was performed using the following protocol: the probe DNA was labelled using the Biotin High Prime kit (Roche, Basel, Switzerland) and detection of hybridization signals was performed using the Vectastain ABC elite kit (Vector Laboratories, Burlingame, CA, USA) and Alexa Fluor 594 Tyramide (Invitrogen). Chromosomes were DAPI stained and the slides were mounted using the VECTASHIELD mounting medium (Vector Laboratories, Burlingame, CA, USA). Chromosomes were screened under an epiflorescence Zeiss Axyoplan microscope; images were captured using an Olympus DP70 digital camera. For the chromosomal location of SSRs on mitotic chromosomes the karyotype description in Willhoeft [22] has been adopted.

### SSR genetic variability estimates and suitability for the assessment of remating

The polymorphic information content (PIC) of each of the 19 SSR loci was determined using the program Cervus 3.0 [23]. For each locus and population, the number of alleles (Na), frequency range, observed heterozygosity (H_O_) and expected heterozygosity (HE) were estimated using the program Genepop version 4 [24]. The same software was also used to test for linkage disequilibrium between pairs of loci in each population (100 batches, 1000 interactions per batch) and for deviations from Hardy-Weinberg (HW) equilibrium, at each locus/population combination, using Fisher's exact test. The Bonferroni correction was used for all tests involving multiple comparisons [25]. The average exclusion probability (Excl.), i.e. the probability of excluding a single unrelated candidate parent from the parentage of a given offspring, knowing the genotype of the second parent, was estimated using the program Cervus 3.0. For each locus and population, the frequency of null alleles was calculated using the Brookfield estimation [26] in Micro-Checker 2.2.3 [27]. For the X-linked loci the number of alleles and the frequency range were evaluated using the data from both males and females, whereas heterozygosity, exclusion tests and frequency of null alleles, were calculated using the data obtained from only the females. Microsatellite Analyser (MSA) software, version 4.05 [28] was applied to determine the degree of genetic differentiation between Kabunkanga and Buvuma in terms of F_st_ [29].

### Reliability of the sperm-typing method

There are three potential sources of errors associated with the genotyping of the sperm stored in the spermathecae [30], [31];

#### a) contamination of the sperm DNA with DNA from the female

If the amount of the contaminating female-derived DNA in the sample is sufficient to be amplified, the number of matings per female may be overestimated. Contamination of sperm DNA by female DNA is assumed when all the female alleles are present in the sperm sample with or without additional alleles. The probability that this sperm genotype pattern is due to a true double mating can be calculated [32]. Even using moderately polymorphic loci, this probability is very low, therefore, when all the female alleles were present at all the loci in the sperm sample, such a pattern was conservatively attributed to contamination.

#### b) the non-detection error

If two or more males share identical genotypes, multiple matings can remain undetected. The probability that two males have identical alleles at all loci (non-detection error) is given by:
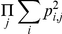
Where *p_ij_* is the population frequency of the allele *i* at the locus *j* [33], [34].

#### c) multiple matings may be underestimated when there is unequal contribution of different males to the total amount of the sperm sample

In this case, because PCR is a competitive process, alleles from the underrepresented male may not be amplified. The sensitivity of our sperm-typing method was tested by mixing serial dilutions of two different sperm DNA samples with known genotypes. This experiment allowed us to determine the minimum relative contribution of sperm form one male (to the total amount of sperm in the spermatheca) detectable in our genotyping assays.

### Detection of remating

Two different approaches were used to determine the minimum number of mates per female. The first is a simple descriptive method, based on direct count, which does not involve any probabilistic model. The second approach, which incorporates information derived from the allele frequency in each population using the Hardy-Weinberg principle, provides expected values of multiple matings. This information would be lost if one followed only the first approach. It is worth noting that the expected values of multiple matings also take into account cases in which both males and females, in the population, share the same alleles for each locus. These cases are not recognizable as rematings in the direct count. For the second approach two different viewpoints were adopted: (a) the maximum likelihood technique and (b) the Bayesian analysis. For elementary explanations of these methods see [35]–[37].

#### Direct count

The count of paternal alleles detected in the stored sperm provided the minimum number of mates for each female. This provides a conservative estimate of the number of mates/female. For sex-linked loci, the maximum number of alleles found in the sperm corresponds to the minimum number of mates, since each male could contribute only one allele to the sperm sample. For autosomal loci, the minimum number of mates was established taking into account that each male could potentially contribute two different alleles. On these bases, the number of matings was estimated for each female and, to obtain an overview of remating in each population, the mean number of matings per female (*N_count_*) was computed.

#### Statistical inference based on probabilistic model

For this second approach, the probability of observing the presence of an array of sperm alleles (*D*), when a given number of males contributed to the sample, was determined on the basis of the allele frequencies. Subsequently, this probability was used a) to obtain maximum likelihood estimators (MLE) of the (unknown) number of mates and b) to obtain a Bayesian estimator of the (unknown) numbers of mates.

For a given autosomal locus *j*, if the number of mates contributing to the sperm sample is *N*, the probability of observing a certain pattern of m different alleles, say *D_j_ = (i_1_…i_m_)*, is
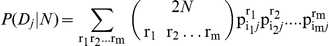
where *p_i,j_* is the allele frequency of the i-th allele at locus *j* and the sum is taken over all vectors *(r_1_, r_2_…r_m_)* such that *r_1_≥1,…, r_m_≥1* and
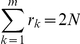



To clarify the expression *P(D_j_|N)* it is enough to note that
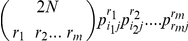
gives the probability of observing r_1_ alleles of the i_1_th type, r_2_ alleles of the i_2_th type and so on, in *2N* trials, under the usual assumptions of independence. For X-linked loci this probability has the same expression with *N* in place of *2N*. Hence, for *J* independent loci the probability of observing a certain pattern of alleles *D = (D_1_,…D_J_)* is
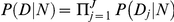



#### a) Maximum likelihood

To obtain a maximum likelihood estimator (MLE) of the number of matings for a given female with a certain pattern of sperm alleles *D*, *P*(*D|N*) was computed for *N = *1,….,35 (i.e. a number sufficiently high to exceed the maximum number of males a female could have mated) and the number *N* with the highest probability *P(D|N)* was chosen. This process was repeated for each female fly in the two considered wild populations. To get a comprehensive view of the population, after computing the maximum likelihood estimates *N_1_^MLE^*, *N_2_^MLE^*,*…. N_K_^MLE^* for each *K* females, it is useful to compute the mean
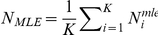
which can be used as an estimate of the mean number of matings in the population.

#### b) Bayesian analysis

Regarding the Bayesian analysis, the main goal was to provide the posterior distribution of the number of mates per female, given the arrays of sperm alleles detected in the population. In order to do this, the numbers of matings associated with each of *K* females in the population, *N_1_,…,N_K_*, were assumed to be independent with a common geometric distribution with unknown parameter *θ*. This is the same as saying that the (conditional) distribution of (*N_1_,…,N_K_*) given *θ* is
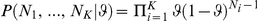



According to the Bayesian paradigm, *θ* is thought of as a random parameter with *Beta (a,b)* prior, that is
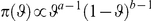

*a* and *b* being positive numbers (hyper-parameters) to be specified later. It must be remembered that the numbers of matings *N_1_, N_2_*,… are not directly observed and, so they must be considered as latent variables. Hence, in order to estimate the number of matings, it is reasonable to evaluate either the conditional distribution of (*N_1_*,*…*,*N_K_*) given the observed alleles or the predictive distribution of matings for an hypothetical new fly. In view of this remark, it is clear that we do not aim at estimating the unknown parameter *θ* of the geometric distribution. It turns out that the conditional distribution of (*N_1_*,*…*,*N_K_*) given the observed data is less sensitive to the prior choice compared to the predictive distribution. For this reason only the results obtained using the posterior expected frequencies of (*N_1_*,*…*,*N_K_*) will be reported. To be more specific, *Obs*, the complete set of patterns of sperm alleles observed in a population of *K* flies, can be summarized as
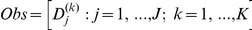
where 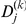 describes the alleles observed in the *j*-th locus of the *k*-th fly. Again, we can assume that, given the number of matings (*N_1_*,*…*,*N_K_*), 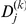 are independent with distribution 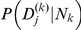 already described. Finally, let *F_n_* be the frequency of *n* (*n = *1,2,…) among the (*N_1_,…, N_K_*), that is
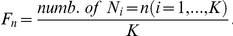



The posterior expected frequency of mating is then
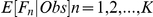
where, as usual, *E* stands for the expectation value. In particular, it is possible to compute the posterior expected number of matings (to be compared with N_MLE_)
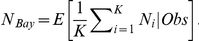



The actual computation for the two sets of data, was carried out by standard Montecarlo simulations.

## Results

### Characterization of the SSR loci

**Figure 2:**
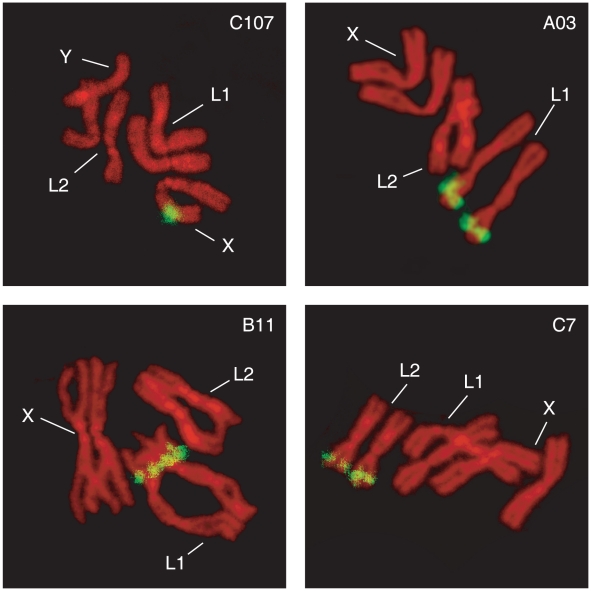
Fluorescence *in Situ* Hybridization (FISH) to *Glossina fuscipes fuscipes* mitotic chromosomes using the microsatellites C107, A03, B11 and C7 as probes. Numbers of chromosomes are as described in Willhoeft [22].

The characteristics of the 19 identified SSR loci, in terms of primer sequence, amplification conditions and PIC values, are summarized in Table 1.The characterization was performed on DNA from single flies (29 females and 20 males) collected in KB. Eleven of these loci are X-linked while the remaining eight are spread along the L1 and L2 autosomes, as assessed by chromosomal *in situ* hybridization analyses (Figure 2). Out of these 19 loci, 4 autosomal (A03, B11, C7, D101) and 2 X-linked (C107 and D3) loci appear to be good candidates for sperm genotyping in remating studies, as they display high PIC values and are easy to score.

**Table 1:** Characteristics of loci used in this study for *Glossina fuscipes fuscipes*.

Locus	Repeat motif	Primer (5′-3′)	Ta (°C)	PIC^d^	Chromosomal inheritance
A03	(CA)_12_	F: AGCCGCTTTAAGTTTGTTGC	54	0.639	autosomal
		R: GTTTGTTCGGTGGGCAATAC			
A06^a^	(AC)_10_	F: TCCCGTCACAGACGTTACAATCAAAGGTGGGTCTATC	50	0.211	X-linked
		R: CCGAACGAATACAGGTAAAG			
A09^a^	(TG)_6_TA(TG)_6_TA(TG)_6_	F: AACTGTTTCTTCAACAAAAATCCA	54	0.099	X-linked
		R: GAAAATCGCGCATATTGCTA			
A112^a^	(CA)_8_	F: TCCCAGTCACGACGTCGTTTCCTCTTCACCTCCAC	56	0.000	X-linked
		R: CGGGCTGTCTTCTTTTGG			
B03	(CT)_18_	F: AGTCCGGGGATTTATTGACC	58	0.416	X-linked
		R: TGGTTGGTACTGATGCGAAA			
B05^b^	(GA)_12_	F: CGCGCTTAGCTAGGAAACTC	58	0.364	X-linked
		R: AACGATTTGCTGTCCTCGAT			
B06	(GA)_18_TA(GA)_2_	F: TCCCAGTCACGACGTAGTGCCAATGAAGAGAGTGTC	50	0.844	autosomal
		R: CCAACTGTTTAGGGCTGTTC			
B11	(GA)_28_	F: TTAGAGCCAGTGCCAATGAA	54	0.866	autosomal
		R: TCAGTGAAGACATATTCGCATGTA			
B109	(GA)_9_	F: CCGAGAGAGTAGCGGAGAGA	58	0.228	X-linked
		R: CCGGCTATGCCCACTTAATA			
C7^c^	(TGA)_18_	F: GAATTTTAACAAATTGGACTTACAG	54	0.696	autosomal
		R: CCAGGTTAAAACCAGTAACTTCC			
C104	(TGA)_7_TGG(TGA)_6_	F: TGTGAGTACTCGGGGATCTCT	52	0.000	autosomal
		R: GGCAGCAACAAGTTTCCATT			
C107^a^	(TCA)_12_	F: TCCCAGTCACGACGTCGCGGCACCTGTTAGTTAGT	53	0.603	X-linked
		R: GGCACGTGAACTAATGCAAA			
D3	(CAG)_9_	F: CTTTAATGGCTTCGCAGGAG	58	0.572	X-linked
		R: TGCGCATTATTTGATGTTGC			
D05	(GCA)_10_	F: TTGGATTAGCAGCACGAATG	58	0.405	autosomal
		R: GGGAATTGGAATTGTGAGGA			
D06^a^	(GCT)_9_	F: TAACGGGGAGCTAAAAGAAG	53	0.064	X-linked
		R: AAATCATCAGCAGCATCATC			
D12	(CTG)_8_	F: ATTCCCATTGCTGGTTGATG	56	0.118	X-linked
		R: ACGGGTTAGCAAATGAAACG			
D101	(TGC)_8_	F: CTCATGCGTCTGCCTTTACA	58	0.639	autosomal
		R:AGGAGCAATGATGTGTTGGA			
D103	(GCTGAT)_7_	F: GCGGTGTTGCTAGTGGTGTA	53	0.434	X-linked
		R: CCAAGACCATTAACCCATGC			
D109^a^	(AGC)_9_	F: TCCCAGTCACGACGTATCTGCCAATGACATGAATATC	59	0.107	X-linked
		R: CAGTTGGTGTCCGTGTGT			

a Brown et al. [21],

b Abila et al. [7],

c Beadell et al. [8],

d PIC was determined in individuals from population KB.

### Pattern of variation of SSR markers in Kabunkanga and Buvuma Island

The variability estimates describing the suitability of the six loci: A03, B11, C7, D101, C107 and D3, for remating analysis in KB and BV, are shown in Table 2. The number of alleles per locus ranged from 6 to 12 with a mean of 8.83 in the KB population, and from 3 to 11 with a mean of 7.00 in the BV population. After Bonferroni correction [25] for multiple comparisons, Fisher's exact test revealed that the six loci are in Hardy-Weinberg equilibrium in both populations. No significant genotypic linkage was detected between the six loci (Fisher's exact test, Genepop) and therefore they can be considered as independent loci. Analyses performed with Micro-Checker [27] indicated that the average frequency of null alleles is low, 0.02 in KB and 0.01 in BV. The accuracy of these six loci for assessing remating is measured by their combined probability of excluding (Excl) an unrelated candidate parent from parentage when the genotype of the mother is known. The combined exclusion value is 0.99 in KB and 0.93 in BV. The different levels of variability between KB and BV populations is accompanied by a significant level of differentiation [38], as the estimate of F_ST_ is equal to 0.174 between the two populations.

**Table 2:** Comparison of variability estimates in Kabunkanga and Buvuma Island.

Population	Sample size (f; m)	Locus	N_a_	Frequency range	PIC	H_O_	H_E_	A_n_	Excl.*
Kabunkanga	29; 20	A03	10	0.01–0.51	0.66	0.65	0.69	0.02	0.48
		C7	7	0.02–0.42	0.70	0.74	0.74	−0.01	0.52
		B11	12	0.01–0.26	0.86	0.74	0.87	0.06	0.72
		D101	6	0.01–0.39	0.65	0.61	0.71	0.05	0.45
		D3	10	0.02–0.51	0.57	0.59	0.62	0.01	0.39
		C107	8	0.01–0.57	0.60	0.64	0.66	0.00	0.42
									**0.99**
Buvuma Island	40; 20	A03	8	0.01–0.55	0.61	0.55	0.65	0.06	0.43
		C7	5	0.01–0.72	0.39	0.47	0.44	−0.02	0.23
		B11	11	0.01–0.49	0.68	0.78	0.72	−0.04	0.52
		D101	3	0.04–0.59	0.41	0.51	0.52	0.00	0.22
		D3	9	0.01–0.53	0.63	0.59	0.67	0.04	0.46
		C107	6	0.01–0.73	0.37	0.35	0.39	0.02	0.22
									**0.93**

N_a_, number of alleles (mean number in the total); Frequency range, the minimum and the maximum allele frequency observed; PIC, polymorphic information content; H_O_, observed heterozigosity (mean H_O_ in the total); H_E_, expected heterozigosity (mean H_E_ in the total); A_n_, frequency of null alleles H_E_−H_O_/1+H_E_ [26] (mean A_n_ in the total).

*Excl., The average probability that the set of loci will exclude an unrelated candidate parent from parentage of an arbitrary offspring when the genotype of the other parent is known. At the bottom is reported the combined-exclusion probability estimate of the six loci.

### Reliability of the sperm-typing method for remating assessment

The six microsatellite loci were successfully amplified from sperm DNA isolated from the spermathecae of 29 KB and 30 BV females.

#### Contamination error

**Figure 3:**
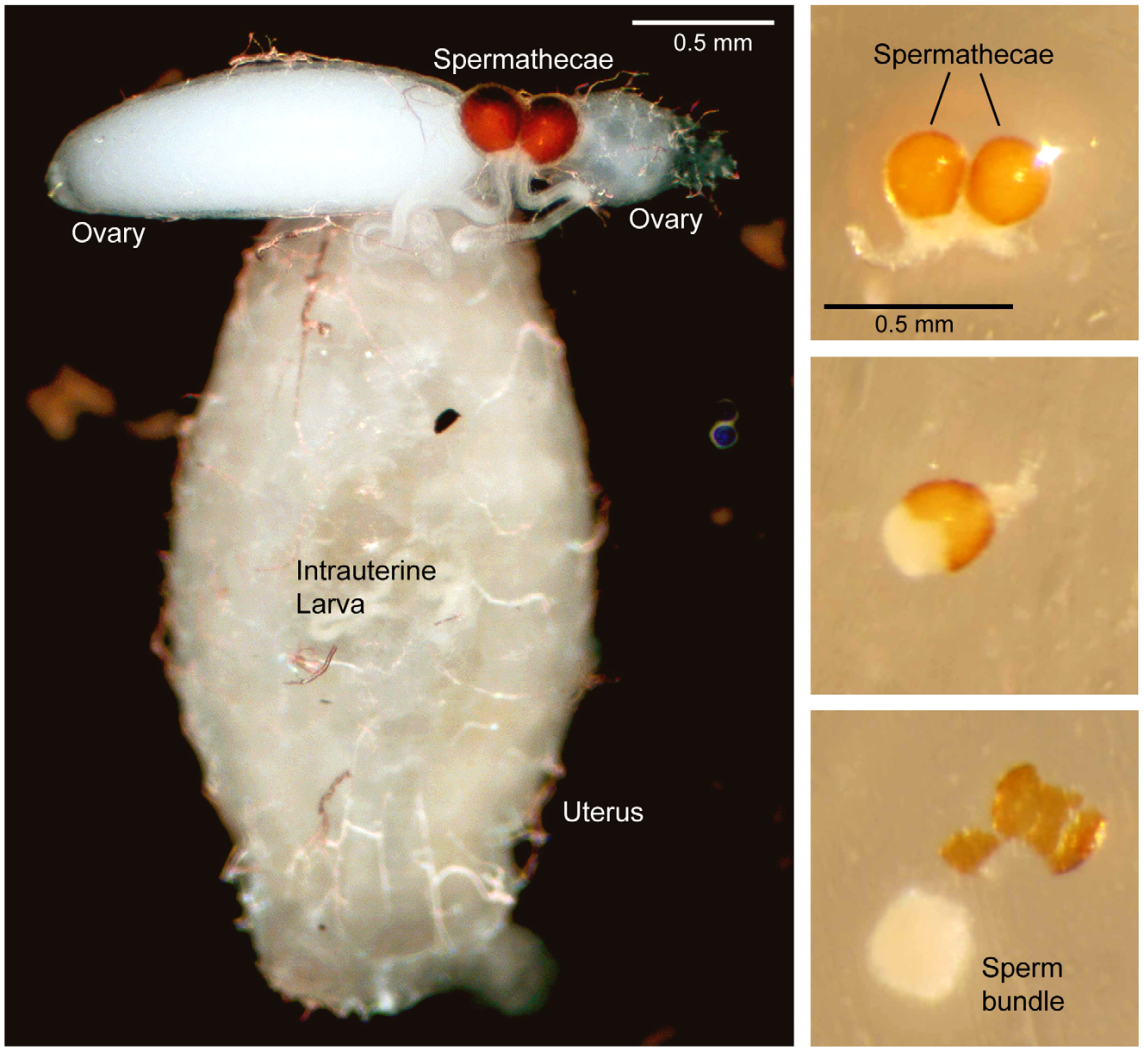
Dissected female reproductive system showing the relative size and location of the spermathecae (Left). Extraction of the sperm bundle from the ruptured spermatheca of a mated *Glossina fuscipes fuscipes* female (Right).

As reliability of the sperm typing method depends on the possibility to exclude contamination of sperm DNA from that derived from maternal tissues, we excluded one case (in population KB) where in the amplified sperm, all the maternal alleles plus four additional alleles at four different loci were present. Most likely this female had mated once as no more than one different (from that of female) allele was present at four loci. The alternative explanation, that the DNA came from two different males, was unlikely as the probability of obtaining this allelic array from a real double mating [32] was very low, 1.9^−7^. In all other cases, in both populations, an occasional overlap of alleles between female and sperm was found only for the commonest alleles and at least one female allele was absent in the sperm. In these cases we assume that all alleles present in the spermathecae were attributable to males. In any case, contamination with maternal tissues is unlikely because of the method used for the isolation of sperm, which are separated from the spermatheca as a compact sperm bundle (Figure 3).

#### Non-detection error

The probability of two random males bearing the same alleles at all loci was low: 0.00026 for population KB and 0.0031 for population BV. Thus, complete overlap of male genotypes cannot be excluded especially in BV, but it is rare and is unlikely to cause a major bias in the remating analysis.

#### Unequal contribution of sperm from different males

Serial dilutions of mixed sperm with known genotype were made to test the sensitivity of our sperm typing method. These trials demonstrated that the rarest sperm genotype was unambiguously detected at a ratio as low as 1∶10. This result is similar to those obtained by Chapuisant [31] and Gretsch and Fjerdingstdad [30] in two different species of ant.

### Detection of remating

#### Direct count

Table 3 shows an example of remating assessment, based on sperm allele score. When contamination can be excluded for at least one locus, the number of mates is equivalent to the number of different X-linked alleles present in the sperm sample and/or to the rounded half of those present at autosomal loci. In other words, remating has been assessed when, in the sperm, more than one allele is present at X-linked loci and/or more than two alleles are found at one autosomic locus. As an example, considering female KB70, the two different alleles (224;227) present in the sperm DNA at the C107 X-linked locus and the three alleles (215; 223;238) at the B11 autosomic locus indicate remating and exclude contamination. The absence of contamination is also assessed by the sperm alleles at A03, with two non-maternal alleles, and at D101, with only one of the two maternal alleles. Applying this method, in the KB population the majority of females (15 out of 28) mated with two males and there was also a single case of triple mating, as revealed by the D3 X-linked locus. Thus, in this population the mean number of observed matings per female (*N_count_*) is 1.61 [(15×2+1×3+12×1)/28] and a random chosen female in this population had mated with more than one male with probability 0.57 (0.53 with two males and 0.04 with three males). In the population from Buvuma Island, 10 out of 30 considered females had mated twice. In this population, the maximum number of matings per female was 2 and the *N_count_* was 1.33 [(10×2+20×1)/30]. Thus, the majority of females in this population had mated with only one male and the probability of remating was 0.33.

**Table 3:** Remating assessment based on sperm allele score.

Population	Female code	Tissue	X-linked loci		Autosomal Loci				No of males
			D3	C107	A03	C7	D101	B11	
Kabunkanga	KB 29	Legs	224-224	224–227	140-140	116–122	172–177	234–238	
		Sperm	**224;227**	227	140;165	116;119	**172;177;179**	210;236	2
	KB 70	Legs	224-224	230–248	163–167	119–125	172–174	210–234	
		Sperm	224	**224;227**	140;146		168;172	**215;223;238**	2
	KB 71	Legs	224-224	224–230	165-165	125-125	170–177	223–236	
		Sperm	224	**218;227**	140;163	116;119	170;177	**215;223;230;236**	2
	KB 72	Legs	209–215	227-227	140–167	116–125	170-170	236–238	
		Sperm	**203;215;221**	**224;227**	140;167	116	168;170	236;238	3
Buvuma Island	BV 33	Legs	224-224	227-227	167–169	116–119	172–177	210–236	
		Sperm	224	227	163	125;128	172	210;250	1
	BV 39	Legs	224-224	227–233	169-169	116–125	172–177	212-212	
		Sperm	224	**212;227**	163	116;125	172	223	2
	BV 87	Legs	227–233	227-227	163–167	125–128	172–177	230–250	
		Sperm	233	227	163	**113;125;128**	**168;172;177**	212;230	2
	BV 89	Legs	224-224	233-233	163-163	116–125	172-172	212–223	
		Sperm	**227;233**	227	**163;169;173**	116;125	172;177	210;212	2

Microsatellite alleles belonging to the 6 loci, two X-linked and 4 autosomal, are identified by size (bp). Sperm alleles are compared with female (legs) alleles. All eight females contain sperm in the spermatheca. In bold are cases that indicate the presence of sperm of more than one male: i.e. 2 or more different alleles at X-linked loci and/or more than two different alleles at autosomal loci. Sperm alleles underscored are cases that eliminate the possibility of maternal DNA contamination.The last column indicates the minimum number of inferred mates.

#### Maximum Likelihood Estimate (MLE)

For each of the two wild populations, the maximum likelihood estimates *N_1_^mle^*, *N_2_^mle^*,*….*, *N_K_^mle^* of number of mates corresponding to the *K* different females of the population, were computed. In the KB population, the estimated number of matings per female ranged from 1 to 3, while in the BV population it ranged from 1 to 2. For the KB population, the MLE estimated mean number of matings/female (*N_MLE_*) was 1.64 and the probability that a female mated with two or three males was 0.57 and 0.04, respectively. This implies that in this population the probability that a female mated with more than one male is around 61%. In the BV population, the estimated mean number of matings per female and the probability of remating were 1.33 and 33% respectively. Thus, these results are consistent with those obtained from the direct count of the sperm alleles.

The statistical significance of the difference between remating frequencies of the two populations was evaluated using the Willcoxon rank sum test. The test shows that the observed difference in remating frequencies between KB and BV is statistically significant (V = 540, P = 0.032).

#### Bayesian estimate

Similar results from both populations were derived using a Bayesian approach. For this method Beta prior with (a,b) = (3,1) was used. Several estimates obtained with different sets of hyper-parameters (data not shown) demonstrated that the choice of (a, b) does not appreciably affect the results. The posterior expected number of matings/female *N_Bay_*, (comparable to *N_count_* and *N_MLE_*), in the KB population is 1.66. The posterior expected probability that a female mates with two males is 0.56 and with three is 0.05, and the overall probability of remating is 0.61. In BV the expected number of matings per female is 1.35. The posterior expected probability that a female mates with two males is 0.34 and with three is 0.004, and the overall probability of remating is 0.35.

**Figure 4:**
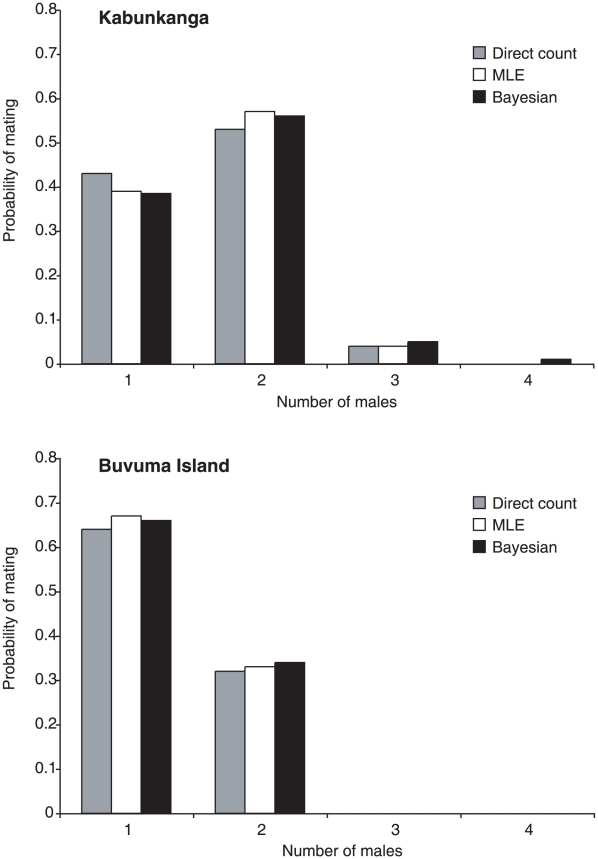
Comparisons of mating and re-mating frequencies of *Glossina fuscipes fuscipes* females in Kabunkanga and Buvuma Island, derived from direct count, MLE and Bayesian estimates.

As shown in Figure 4 and Table 4, the three types of remating estimates are congruent, and confirm a substantial difference between the two populations, as a greater remating frequency (also with more than two mates) was observed and predicted in the most genetically variable KB population.

**Table 4:** Remating assessment in the two populations based on direct count, maximum likelihood and Bayesian estimates.

Population	No females tested	Direct count		Maximum likelihood estimate (MLE)		Bayesian estimate	
		Mean No of matings (*N_count_*)	Probability of remating	Mean No of matings (*N_MLE_*)	Probability of remating	Mean No of matings (*N_Bay_*)	Probability of remating
Kabunkanga	28	1.61	0.57	1.64	0.61	1.66	0.61
Buvuma Island	30	1.33	0.33	1.33	0.33	1.35	0.35

## Discussion

From the 19 analyzed microsatellites, we chose the six most informative loci to estimate the number of matings per female, through the analysis of sperm preserved in the spermathecae in samples from two Uganda populations. The chosen loci are polymorphic for a large number of alleles, which differ in repeat number, making them easy to score for sperm genotyping. The loci are spread over the autosomes L1, L2 and the X chromosome of *G. f. fuscipes*, therefore they assort independently and no linkage disequilibrium has been assessed, providing further statistical power for the mating/remating analyses. The use of X-linked loci in association with autosomal loci provided a more sensitive estimate of number of matings, increasing, for instance, the power of identifying cases of triple matings as shown in Table 3. The direct count of remating estimates has been compared to probability estimates obtained with two inference methods, incorporating population allele frequencies. The mean number of matings per female, obtained from sperm genotyping in Kabunkanga and in Buvuma (Ncount = 1.61 and 1.33 respectively), were very similar to both probability estimates (Table 4), confirming that the use of the six microsatellites did not result in an under-estimation of remating events.

### *Glossina fuscipes fuscipes* is a polyandrous species

Our deductions are based on molecular data, which provide information on the number of males that were able to transfer sperm in a PCR-detectable quantity to a female's spermathecae. Consequently, a conservative (minimum) estimate of the number of males with which a female had mated, was determined in the Kabunkanga and Buvuma wild populations. Although our conditions were able to detect the presence of a second male sperm at a ratio as low as 1∶10, an undetected sperm contribution cannot be excluded. Furthermore cases of failure of sperm transfer, apparently after normal copulations, have been reported [39], [40].

Our results provide the first direct evidence that remating is a common event in the wild and what is more, females of *G. f. fuscipes* may store sperm from different males. These are biologically relevant data for interpreting the reproductive biology of this tsetse species, as it appears that many females preserve sperm from different mates, that could potentially be used for insemination. It is also known that this fly is able to maintain the sperm alive for long time [41]. The simultaneous presence of sperm derived from each mating suggests that one of the potential mechanisms of cryptic female choice, such as sperm displacement, [42]–[45] is not operating in this species. On the other hand, the storage of sperm from more than one male generates the opportunity for sperm competition for fertilization. Whether post-copulatory specific events/mechanisms are operating in the female storage organs to control or drive sperm use, is an important open question, which may clarify how the copulations are translated into fertilization in this fly. It is noteworthy that in *G. austeni* twice mated females utilize sperm from both matings for fertilization of oocytes [10]. If this is the case also for *G. f. fuscipes*, considering the high frequency of remating, this sperm use by polyandrous females may have a strong impact on the effective population size of the population.

### Differences between Kabunkanga and Buvuma

Both direct count estimates of remating and probability estimates, obtained with the two inference methods, are significantly lower in Buvuma than in Kabunkanga: more than fifty per cent (57%) of females mated more than once in Kabunkanga while a smaller proportion (33%) remated in Buvuma. Various factors, which may be interrelated, could be responsible for the observed difference. First, the lower genetic variability in Buvuma, with respect to Kabunkanga, diminishes the discriminatory power of the six SSR loci in this island population, as revealed by the lower combined exclusion probability estimate (Excl 0.93 versus 0.99). Probably this observation is not related to the choice of loci, as Beadell et al. [8] demonstrated that in Uganda there is a significant decline of microsatellite allelic richness from West to East: Kabunkanga and Buvuma are located at a great geographic distance in the West and East, respectively, of the predicted range of the species (Figure 1). Thus, considering that the Excl estimate is related to the level of genetic variability, with an Excl value of 1.00, we would have increased our remating estimates, obtaining an expected value of 0.58 for Kabukanga and 0.36 for Buvuma. Since there is still a difference in the remating frequencies between the two populations, other interrelated eco-geographic and demographic factors must account for the difference. The average age structure may have played a role. In Buvuma Island, flies were caught in April, at the beginning of the rainy season when the population was expanding as also confirmed by the high fly density in the traps, which is about four times greater than the density in Kabunkanga. The Kabunkanga flies were collected in November, at the end of the cooler dry season, when the population undergoes seasonal demographic contractions with a high level of mortality particularly among the young teneral flies while the remaining flies concentrate in moist refugia. In the absence of objective observations regarding the age, such as ovarian measurements and wing-fray analysis [18], we can speculate that in an expanding population, such as Buvuma, the proportion of young flies may be greater than that in a residual population after a seasonal bottleneck, such is the case of the Kabunkanga sample [9], [46]–[51]. It is a reasonable assumption that the surviving flies collected in Kabunkanga at the end of the dry season, had more time and opportunity to remate, than those from Buvuma. In addition, according to Abila et al. [12], male mating competitiveness increases with age, i.e. older males copulate significantly more frequently than younger flies and the peak of female receptivity is between the 8th–13th day after emergence [52]. It has been also reported that *Glossina* females tend to mate more than once with no apparent difference in receptivity and the number of matings appears to be directly related to the amount of semen in the spermathecae: young females contain less semen than older ones [53]. On the basis of these observations, it can be speculated that a demographic parameter such as age could be the cause of the observed difference in remating frequency between Kabunkanga and Buvuma. However, this hypothesis must be confirmed by appropriate analyses. Finally, as the two study sites, Kabunkanga and Buvuma island harbour well established populations which show a significant level of genetic differentiation (Fst = 0.174), we cannot exclude that the distinct genetic backgrounds of the two populations had an effect on the extent of the observed remating estimates.

### Polyandry and tsetse control

Several considerations concern the applied aspects of the present findings. As the Sterile Insect Technique (SIT) is being entertained for tsetse population control, the presence of remating and the fact that females maintain sperm from different mates, potentially available for insemination, may constitute a critical factor for the success of eradication programmes. Although specific experiments would be necessary to assess the sperm use and the possible presence of paternity skew in populations, multiple mating may potentially help maintain genetic variability and increase the effective population size. Thus polyandry may affect the long-term stability and effective size of *G. f. fuscipes* populations. In cases of eradication programmes, re-infestation of cleared areas and/or in cases of residual populations, the occurrence of remating may, unfortunately, enhance the reproductive potential of the re-invading propagules in terms of their effective population size. The comparison of two populations highlights another important factor, which, if confirmed, influences the remating frequency, i.e. the population age structure. Consequently, any vector control programme for *G. f. fuscipes*, according to the present results, must address the greater dimension of the young expanding population in the early wet season, and the increased rate of remating of the fewer, remaining adults after the bottleneck in the dry season. For instance in the case of SIT, a large number of sterile males should be released, also in a population with a reduced number of individuals because of the high rate of remating. These considerations agree with the recommendation to release aged, more competitive, sterile males in all cases [12].

Finally, analyses have identified the presence of parasitic *Wolbachia* infections in some individuals of natural populations of *G. f. fuscipes*, including those from Uganda described here. As it has been suggested that *Wolbachia*-associated incompatibilities may promote polyandry [54], future studies can now investigate the potential influence of *Wolbachia* in the remating phenomenon described here. As *Wolbachia* infections are entertained as a tool to drive genetically desirable phenotypes into natural populations [55], female mate choice and remating may also have an impact on strategies of population replacement.
